# Warming Causes a Decline in Baltic Sea Coastal Sediment Microbial Abundance

**DOI:** 10.1111/1462-2920.70256

**Published:** 2026-02-19

**Authors:** Laura Seidel, Songjun Li, Shahinez Hanna‐Elias, Iryna Rula, Louise Ahlberg, Anders Forsman, Samuel Hylander, Marcelo Ketzer, Mark Dopson

**Affiliations:** ^1^ Centre for Ecology and Evolution in Microbial Model Systems (EEMiS), Linnaeus University Kalmar Sweden; ^2^ Biology and Environmental Sciences, Linnaeus University Kalmar Sweden

**Keywords:** 16S rRNA gene, brackish, live/dead, methane, propidium monoazide, qPCR

## Abstract

Long‐term ocean warming impacts the marine environment, and these effects will be exacerbated by future climate change affecting, e.g., biogeochemical processes and microbial communities. However, how the sediment microbial cell abundance and live/dead ratio respond to warming is poorly understood. In this study, sediment core samples were collected from a Baltic Sea bay artificially heated on average 5°C for > 50 years above a nearby (control) bay unaffected by the heating. Contrary to the expected increased productivity in the heated bay, qPCR‐based sediment cell abundances showed decreased cell numbers along the sediment depth gradient in the heated bay compared to the control bay. This could reflect that a portion of the cells' metabolic energy was diverted to a heat related stress response rather than being used for replication. In addition, live/dead cell ratios showed no clear differences in either bay suggesting the majority of the cells were alive. Finally, sediment depth gradient 16S rRNA gene sequencing confirmed previous studies, showing that prolonged warming shallows sediment biogeochemical zones and related microbial communities. In conclusion, future climate change related warming will likely decrease microbial cell abundances that form part of the food web base, potentially impacting the entire ecosystem.

## Graphical abstract

Long‐term warming in the Baltic Sea coastal area has led to a decrease in sediment microbial cell abundance, alongside a shallowing of sediment biogeochemical zones and associated microbial communities. These changes may become more pronounced in marine sediments under future climate change‐related warming.

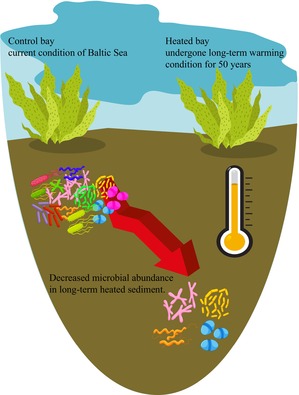

## 1 Introduction

Marine coastal ecosystems are among the most valuable areas on Earth and despite covering just 2% of the ocean floor, they act as major carbon sinks (Mcleod et al. 2011). Due to the more rapid heat transfer through the shallower water column, coastal ecosystems particularly suffer from anthropogenic stressors and are facing increasing pressure due to climate change‐related warming (Cooley et al. 2023). This will likely lead to a decrease in the capacity of the coastal sediment to act as a carbon sink (Pendleton et al. 2012; Lovelock et al. 2017; Deb and Mandal 2021). However, if and to what degree coastal sediments may become greenhouse gas sources remains unclear.

Microorganisms drive global biochemical processes linked to the carbon, nitrogen and sulphur cycles (Turley 2000; Burdige and Komada 2011), making them an important factor in the ecosystem's equilibrium. Microorganisms in marine sediments, especially bacteria, are involved in these different biogeochemical cycles occurring at different sediment depths owing to progressive porewater chemistry changes below the seafloor (Berner 1981; Parkes et al. 1994; Torsvik et al. 1996; Nealson 1997). While, for example, Cyanobacteria sequester atmospheric carbon (Rae et al. 2013), other microorganisms within sediments use various electron acceptors to degrade organic matter. Furthermore, Archaea groups related to methane generation are more predominant below the sulfur‐methane‐transition‐zone (SMTZ) where sulfate concentrations are nearly zero (Wehrmann and Riedinger 2016). Therefore, competition among microbial species alters along the depth gradient from oxic to anoxic conditions (Burdige 2006). It is estimated that 75% of the ocean's total microbial biomass is in the upper 10 cm of sediment at the sea bottom (Turley 2000), while the amount of intact ‘alive’ bacterial cells decreases the deeper the samples are taken in the sediments (Parkes et al. 1994; Kallmeyer et al. 2012). However, how, for example, a warming induced increased microbial replication rate (Clarke and Fraser 2004) may cause a cascade of consequences for organic matter mineralization and the rest of the ecosystem is unknown.

The Baltic Sea is a semi‐enclosed brackish water body (Segerstråle 1969). Due to global warming, the Baltic Sea's temperature has increased most within all marginal seas (Belkin 2009) with an estimation of +0.59°C/decade from 1990 until 2018 in the northern area (Siegel and Gerth 2020) and between 0.03°C and 0.06°C/decade from 1856 until 2005 in the southern region (Kniebusch et al. 2019). This study utilises a Baltic Sea Bay that has been artificially heated for over 50 years by cooling water from a nearby nuclear power plant in Oskarshamn, southeastern Sweden. This bay has been used as a natural laboratory to study the effects of long‐term increased temperatures in natural settings (Seidel, Ketzer, et al. 2022). Additionally, a nearby control bay is used to compare differences between the artificially heated bay and a system unaffected by the powerplant heating. The two bays are directly connected to the Baltic Sea with approximately 1.5 km distance between them (Seidel, Ketzer, et al. 2022). Previous results from this study site show that long‐term warming potentially leads to a decrease in benthic microbial diversity coupled with a broader thermal tolerance and weakened response to temperature fluctuations (Seidel, Broman, et al. 2023). Furthermore, exposure to prolonged temperature rises results in increased RNA transcripts related to stress, suggesting that even after > 50 years of exposure the microbial community has not adapted to the warming (Seidel, Broman, et al. 2023). However, a shallowing in sediment geochemical layers has led to an increase in near‐seafloor bacterial diversity. Similar results were observed at greater sediment depths (up to 20 cm) with higher sulfate fluxes, shallowing the SMTZ (Seidel, Sachpazidou, et al. 2023). Although climate change warming related shifts in the relative abundance of microbial diversity and activity are known, few studies address the effect of warming on actual cell abundance that allow a more detailed understanding of how future climate related warming will affect the coastal environment.

In this study, 16S rRNA gene‐based live/dead measurements were coupled to enumeration of cell numbers to investigate if the shallowing of the redox layers alters the quantitative distribution and live/dead ratio of bacteria and archaea cells with sediment depth. The hypothesis tested was that climate‐related warming increased microbial production and thus elevated cell abundances in the heated compared with the control bay, and with the greatest effect at the shallowest sediment layers.

## 2 Methods

### 2.1 Field Sampling

**FIGURE 1:**
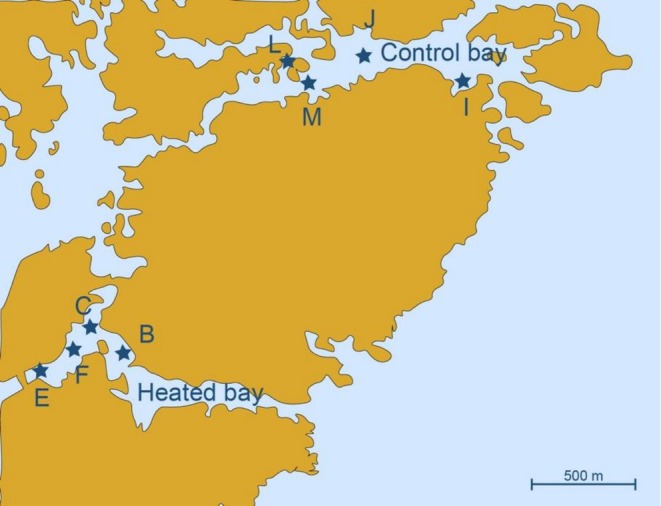
Map of all the sampling sites at the heated and control bays sampled in this study. The map was adapted from Seidel et al. (2024).

Field sampling was carried out on 29 to 30 March and on 6 April 2023 at two coastal bays north of Oskarshamn, southeastern Sweden (Figure 1). One bay had been artificially heated for > 50 years by the discharge water from a close‐by nuclear power plant (referred to as the ‘heated bay’; 7.421750N, 16.666967E) while the second bay was unaffected by the discharge (referred to as the ‘control bay’; 7.433517N, 16.683700E). The two bays have been extensively described in previous publications (Seidel, Broman, et al. 2022; Seidel, Ketzer, et al. 2022; Li et al. 2024). Bottom water temperature, pH, and salinity were measured in situ using the MultiLine Multi 3620 IDS/3630 IDS multimeter from WTW, Xylem (Washington DC, USA) with the Sentix pH electrode IDS 940‐3 and the TetraCon conductivity cell electrode 925 for the salinity from Xylem (Washington DC, USA). Sediment cores were collected at four different sampling sites (Figure 1) within each bay using a HTH Pylonex gravity corer (Ume, Sweden; outer diameter 70 mm, inner diameter 66 mm). The cores were aseptically sliced at six different sediment depths (0–1, 1–2, 5–6, 8–10, 15–17, and 22–24 cm) and collected in sterile 50 mL Falcon tubes, except for site B where the sediment core was too shallow to collect the 22–24 cm layer (File S1). Each sample from every site and depth was treated and analysed individually while certain statistical analyses and visualisations used average values across replicates. Subsamples of the collected sediment were taken as previously described (Broman, Sjöstedt, et al. 2017) but in short, acid‐washed 15 mL Falcon tubes and sterile 2 mL Eppendorf tubes were filled for chemistry and organic matter analyses, respectively. The samples for chemistry analysis were cooled while being transported back to the laboratory on the same day where they were stored at −20°C until analysis.

### 2.2 Propidium Monoazide Identification of Live/Dead Cells

The microbial cell live/dead analysis used propidium monoazide (PMA) that penetrates cells with a compromised membrane and irreversibly binds to the cell's DNA after UV irradiation such that subsequent PCR amplification is inhibited and thereby, only ‘live’ cells are amplified (Desneux et al. 2015). The analysis was immediately performed in the field by filling two sterile Eppendorf tubes (1.5 mL) with 250 mg of sediment and mixed with sterile 800 μL PBS (phosphate‐buffered saline) plus 12.5 μL distilled water in one tube (without PMA, referred to as PMA(−)) and 12.5 μL of PMA in the second tube (final concentration 50 μM PMA, referred to as PMA(+)). The PMA(+) and PMA(−) Eppendorf tubes were immediately incubated for 10 min in the dark and at ambient temperature and thereafter for 15 min under blue light (400–470 nm wavelength) using the PhAST Blue‐Photo activation system for tubes (GenIUL, S.L., Spain). During the incubation time, the samples were mixed to ensure the PMA reached all cells. After the incubation, the tubes were flash frozen using liquid nitrogen before being transported to the laboratory on the same day and stored at −20°C until further analysis.

### 2.3 Geochemical Analyses

Geochemistry measurements used the porewater from the collected 15 mL Eppendorf tubes. The tubes were centrifuged for 15 min at 2200 × *g* and the supernatant extracted and transferred to a new acid washed 15 mL Falcon tube. Parameters were measured using LCK cuvette kits with a DR 3900 spectrophotometer (both Hach, Colorado, USA): nitrite (LCK341), nitrate (LCK339), iron (LCK320), sulfate (LCK353), ammonium (LCK303), and phosphate (LCK350). Porewater dissolved organic carbon content was measured by filtering the samples through SYR FIL glass microfiber 0.7 μm filters (Thermo Scientific, Gothenburg, Sweden) and measured with the total organic carbon cuvette test (LCK381).

### 2.4 DNA Extraction

DNA extractions from the PMA(−) and PMA(+) samples used the DNeasy PowerSoil Pro Kit (Qiagen, Hilden, Germany) according to the manufacturer's guidelines except that the DNA samples had a final elution volume of 50 μL. DNA concentrations and extraction purity were measured using a Qubit 2.0 (Invitrogen, Göteborg, Sweden) and NanoDrop 2000 (Thermo Scientific, Göteborg, Sweden), respectively. The extracted DNA was stored at −20°C until further use.

### 2.5 qPCR Amplification

To perform qPCR analysis, bacterial and archaeal standards were created for each primer set with *Escherichia coli* using the forward primer 908F and reverse primer 1075R (Ohkuma and Kudo 1998) to create a 167 bp product and *Ferroplasma acidiphilum* BRGM4 using the forward primer 915F and reverse primer 1059R (Kubo et al. 2012) to give a 144 bp product. The PCR program for the standards was: initial denaturation at 98° for 30 s; 30 cycles of denaturation at 98° for 10 s, annealing at 60° for 30 s, and extension at 72° for 20 s; and a final extension at 72°C for 5 min. Subsequently, the qPCR products were cleaned using the AMP Pure XP PCR clean up kit (Beckman Coulter) and the concentration measured in triplicates as described above and tested on an agarose gel to confirm the correct fragment size. To use the standard within the qPCR analysis, a 10‐fold dilution series was performed with DNAse/RNAse free water until 10^9^ dilutions (start concentration 28.83 and 5.75 ng/μL for bacteria and archaea, respectively) and DNAse/RNAse free sterile water was used as a negative control (*n* = 3). The standard was always kept on ice while handling and immediately put back in the freezer at −20°C after taking the required volume to maintain the DNA integrity.

qPCR analysis was conducted on the LightCycler 480 Multiwell machine (Roche Diagnostics, Solna, Sweden). For the bacteria and archaea primers, the reaction solution contained 5 μL of MasterMix (PowerUp SYBR Green Master Mix), 0.2 μL of forward and reverse primer (Bacteria 908F and 1075R (Lever et al. 2015) & archaea 915F and 1059R), and 3.6 μL of DNAse/RNAse free H_2_O (protocol: incubation at 50°C for 2 min, initial denaturation at 95°C for 10 min, then denaturation at 95°C for 15 s, annealing at 60°C for 30 s, and extension at 72°C for 45 s for 45 cycles, followed by a final extension at 80°C for 5 s). For each bacteria sample, a dilution of 1:50 as well as 1:100 and for each archaea a dilution of 1:10 was added in duplicates. To obtain the copy of gene per g sediment dry weight, tubes collected for organic matter measurement were dried in an oven for 4 days at 50°C coupled with the sample gene copy numbers based on the standard curve (described above). The standard curve linear trend line, equation, *R* ^2^, and the slope calculated to check if *R* ^2^ was > 0.990. The efficiency was then calculated based on the equation: efficiency = (10^(−1/slope)^−1) × 100 (varying between 88% and 98%). The sample gene copy numbers were calculated using the equation ‘gene copy number/μL = DNA conc. (g/μL) *AVOGADRO constant/M(Fragment)’ and then converted to gene copy number per g. Cts value differences between replicates threshold was < 0.5 while outliers > 0.5 were excluded from the calculations.

### 2.6 16S rRNA Gene Amplification and Sequencing

The 16S rRNA gene amplification for bacteria and archaea communities used PCR primers 341F‐805R (Hugerth et al. 2014) and 517F‐958R (Dutta et al. 2019), respectively. Amplification of 16S rRNA gene amplicons as well as sequencing library preparation were done as previously described (Lindh et al. 2015). Both bacteria and archaea 16S rRNA gene amplicon sequencing (2 × 301 bp pair‐end setup) was performed on the Illumina MiSeq platform by Science for Life laboratory (SciLifeLab), Stockholm, Sweden.

### 2.7 Bioinformatics

The raw sequencing data were analysed with the nf‐core ampliseq pipeline (v. 2.8.0) within Nextflow (v. 23.10.1) using the default settings (Straub et al. 2020). In short, the data were preprocessed (primer removal with cutadapt (v. 3.4)) and then quality filtered, post processed, and taxonomical annotated using DADA2 (v. 1.28.0; (Callahan et al. 2016)) to generate amplicon sequence variants (ASVs). The taxonomical classification was performed using the GTDB (Genome Taxonomy Database, release R08‐RS214.1 (Parks et al. 2018)) for bacteria sequencing data. The SBDI‐GTDB (Sativa curated 16S GTDB database—Release R07‐RS207‐1) was used for the archaeal targeted sequencing data.

### 2.8 Data Analysis and Statistics

All data were analysed using R (v. 4.4.0) within RStudio (v. 2024.04.0) with the used packages stated below and further information can be found within the R code provided in the Data Availability Section. To understand the relationship of the response variable with the depth gradient and how it differed across the bays, a mixed linear model was used to test for differences using the ‘lmer’ function within the lme4 (v. 1.1‐35.3; (Bates et al. 2015)) and lmerTest (v. 3.1‐3; (Kuznetsova et al. 2017)) packages using depth*bay (interaction) as fixed effects and the sampling site as random effect (settings, REML = TRUE).

The qPCR copy numbers were combined with the sediment dry weight measurements to give 16S rRNA gene copy numbers/g sediment dry weight for the PMA(+) and PMA(−) bacteria and archaea amplicons. Afterwards, absolute cell numbers/g dry weight based upon bacteria and archaea families were calculated by multiplying the percentage relative abundance from the 16S rRNA gene amplicon sequencing by the gene copy numbers obtained for the respective bacteria and archaea from the qPCR analysis. The gene copy number data as well as the ASVs were tested for potential differences between the PMA(+) and PMA(−) within each bay at the different sampling depths. The gene copy numbers were tested for normal distribution using the ‘shapiro.test’ function within the stats package (v. 4.4.0). As the data for bacteria and archaea were not normally distributed and dependent, a Wilcox test was conducted (rstatix v. 0.7.2) to test the cell abundance differences between the heated and control bays. Furthermore, a paired Wilcox test was used to investigate significant differences between the PMA(+) and PMA(−) treatments. No statistically significant differences from the PMA treatment were observed within the data sets and therefore, the PMA(+) and PMA(−) data were combined for further sequencing analysis steps.

Rarefaction curves (File S2) were conducted within vegan (v. 2.6‐6.1, settings step size 20) that suggested the majority of the microbial diversity was identified. Bacterial and archaeal alpha diversity was analysed in the vegan package using the normalisation method of scaling with ranked subsampling (SRS package v. 0.2.3, default settings, (Beule and Karlovsky 2020)) and presented in Supplemental File S2. Mixed linear model (lme() function within nmle package, v. 3.1‐164) with depth, bay, and PMA (treatment) as interaction fixed effects, and the factor ‘site’ as random effect using the method ‘REML’ was used in an ANOVA to test for potential differences in the different depth layers of the sediment of the bays and the PMA treatment on the diversity of the communities (File S3). The ANCOMBC2 R package (v. 2.6.0) was used for the differential abundance analysis of archaea and bacterial communities at family level. The dunnest test was used to compare sediment depths to the surface part (0–1 cm) in each bay with Holm‐Bonferroni method to adjust *p*‐values from multiple comparisons.

Finally, a genomic prediction of potential gene function was conducted using the tool PICRUSt2 (Douglas et al. 2020). PICRUSt2 was used to predict microbial metabolic pathways (default settings) of the bacteria and archaea 16S rRNA gene sequence ASVs. The output data ‘predicted metagenome’ with the KEGG Identifiers was used to run differential abundance analysis using the ‘ancombc2’ function (ANCOMBC package v. 2.6.0, fix_formula: ‘depth’, p_adj_method: ‘holm’, alpha: 0.05, pairwise: TRUE, dunnet: TRUE (Lin and Peddada 2024)). Multiple pairwise comparison against different sediment layers compared to the surface sediment layer (0–1 cm) in each bay was run using the Dunnett's test (Files S4 and S5). Significant differential abundant KEGG Orthology (KO; see https://www.genome.jp/kegg/) identifiers between the tested groups were selected and filtered for the nitrogen, sulfur, and methane cycling genes and a heat map constructed using the average relative abundance of the predicted genes.

## 3 Results

### 3.1 16S rRNA Gene Copy‐Based Live/Dead Cell Numbers

**FIGURE 2:**
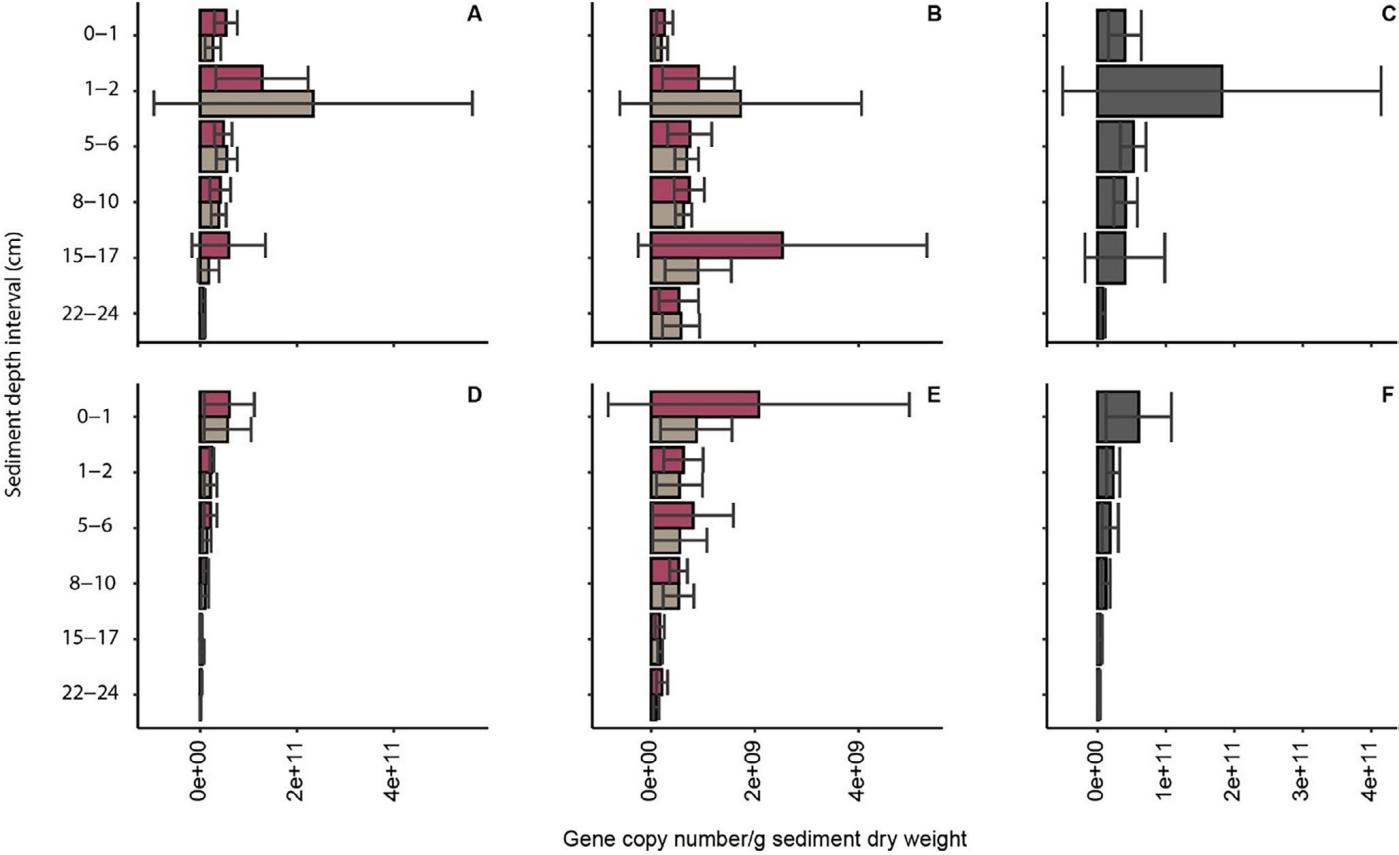
Control (A–C) and heated (D–F) bay qPCR‐based 16S rRNA gene copy number cell numbers for the bacteria (A and D) and archaea (B and E) in the presence (burgundy) and absence (grey) of PMA. Combined data for bacterial and archaeal PMA(+) and PMA(−) results are provided in (C and F, respectively). The data represent biological replicates (*n* = 4 for each bay in (A, B, D, and E) and *n* = 8 for totals in each bay (C and F)) with SDs.

The live/dead ratio of sediment microbial cell abundance was estimated by comparing the results from the PMA(+) and PMA(−) treatment. Differences between the PMA(+) and PMA(−) treatment for the bacteria summed for all tested depths were insignificant in both the heated bay (Wilcox test, *n =* 23, effect size = 0.17, *p =* 0.46) and control bay (Wilcox test, *n =* 24, effect size = 0.29, *p =* 0.17; Figure 2) suggesting that all the cells were alive. Furthermore, there were no significant differences between the bacterial PMA(+) and PMA(−) treatments at each individual tested depth in either bay (*n =* 4 per treatment group/depth/bay, *p* > 0.1). In addition, a similar test for PMA(+) and PMA(−) treated archaeal cells at all depths was also insignificant in the heated bay (*n =* 23, effect size = 0.33, *p =* 0.12) and control bay (*n =* 24, effect size = 0.18, *p =* 0.39). Finally, there were no significant differences between the archaeal PMA(+) and PMA(−) treatments at each individual tested depth in either bay (*n =* 4 per treatment group/depth/bay, *p* > 0.1). Therefore, the PMA(+) and PMA(−) values were merged in the further analyses.

Combining the qPCR measurement of the bacteria and archaea 16S rRNA gene copy‐based cell numbers for all depths within the heated versus control bays gave significantly higher cell numbers in the control bay compared to the heated bay (Wilcox test, *n =* 94, *p* < 0.01; Figure 2). In addition, comparing the cell number depth trends in the heated bay showed the highest bacterial (5.8 × 10^10^ ± 4.6 × 10^10^ gene copy numbers/g dry weight) and archaeal (1.5 × 10^9^ ± 2.1 × 10^9^ gene copy numbers/g dry weight) counts within the first 0–1 cm of the sediment layers. These bacterial and archaeal cell numbers steadily decreased with depth to 2.0 × 10^9^ ± 1.1 × 10^9^ and 1.5 × 10^8^ ± 9.8 × 10^7^, respectively in the 22–24 cm sediment layer. In contrast, the control bay bacteria and archaea cell counts were low between 0 and 1 cm, peaked with 1.8 × 10^11^ ± 2.3 × 10^11^ and 1.3 × 10^9^ ± 1.6 × 10^9^ between 1 and 2 cm, before steadily decreasing with depth for bacteria and maintaining an approximate even number for the archaea.

### 3.2 Quantitative Analysis of 16S rRNA Gene Based Community

**FIGURE 3:**
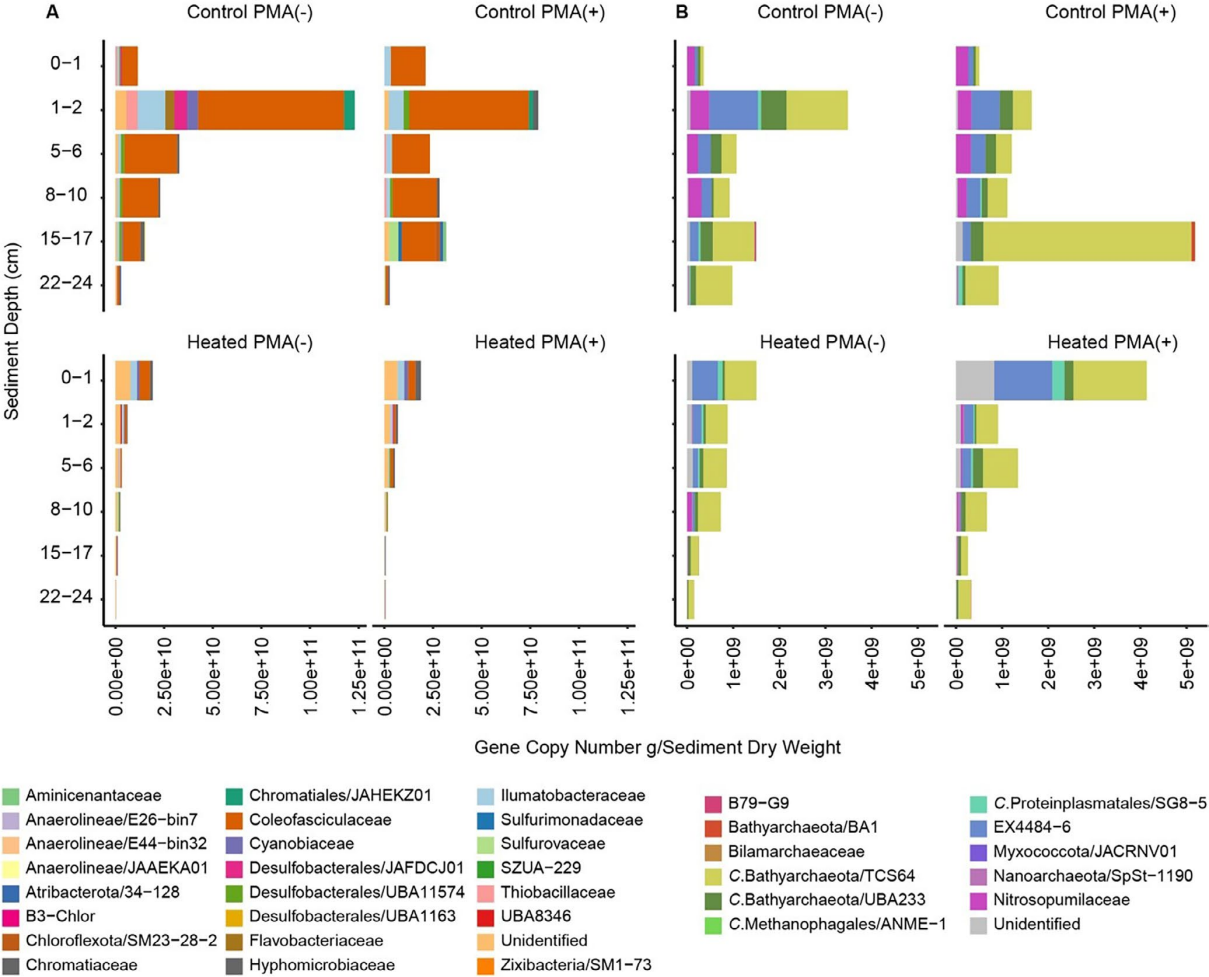
Relative abundance of the heated and control bay 16S rRNA gene bacteria (A) and archaea (B) family level ASVs in the presence and absence of PMA. The relative abundances were overlaid on the qPCR‐based cell numbers to give absolute cell numbers for each taxon.

Combining the 16S rRNA gene relative abundances with the qPCR‐based cell numbers gave absolute values for the individual taxa in the two bays (Figure 3). Irrespective of the addition of PMA, the control bay 16S rRNA gene ASVs were attributed to the bacterial families Coleofasciculaceae, Illmatobacreaceae, Thiabacillaceae, and JAFDCJ01 (Desulfobacterales). Similarly, the heated bay 16S rRNA gene ASVs were attributed to the bacterial families Hyphomicrobiaceae, B3‐Chlor, and UBA8346. These families were less abundant in the heated bay with for instance, a sharp decrease in cell numbers with increasing depth, leading to a decrease of Illumatobaceraceae being detected within the first 2 cm (3.5 × 10^9^ and 1.4 × 10^9^ gene copy numbers g/dry weight) and declining towards deeper depths (e.g., 5.3 × 10^7^ at 15–17 cm).

Once again irrespective of the addition of PMA, the control bay archaea TCS64 (*Candidatus* Bathyarchaeota) family cell abundance was identified from 1 cm sediment depth and below (Figure 3). In addition, the EX4484‐6 (Thermoplasmatota) family was detected with peaks in the 1–2 cm layers (1.1 × 10^9^ gene copy numbers g/dry weight) and declined in the layers 5–6 cm (2.8 × 10^8^), 8–10 cm (2.2 × 10^7^), and 15–17 cm (1.7 × 10^7^). Similar results were observed for the heated bay abundances that were dominated by the TCS64 (*Ca*. Bathyarchaeota) and EX4484‐6 (Thermoplasmatota) families.

### 3.3 Differential Abundance of 16S rRNA Gene Based Community With Sediment Depth

**FIGURE 4:**
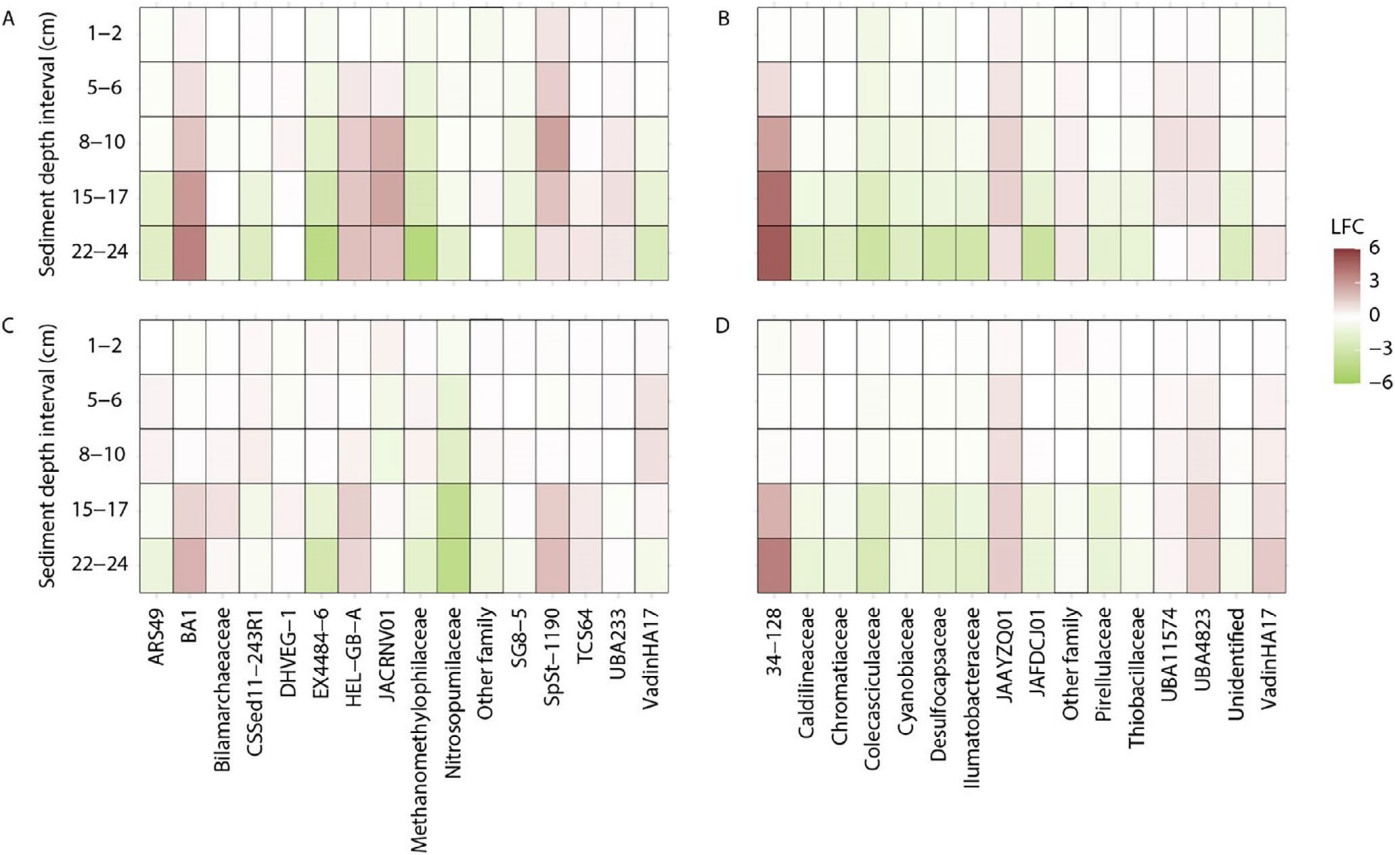
16S rRNA gene amplicon sequence variant differential relative abundance analysis (at the level of family) along the depth gradient for the archaeal (A) and bacterial (B) heated bay samples together with the archaeal (C) and bacterial (D) control bay data. The data represent the top 15 most abundant families with statistically significant differential abundances (*n* = 4 for each bay) based upon the 0–1 cm sediment data as reference. The scale represents the log fold change (lfc) value from the analysis showing red colours higher relative abundances in the deeper layer compared to the 0–1 cm layer, while the green colours show lower relative abundances in the deeper layers compared to 0–1 cm.

The differential abundance analysis showed the microbial communities contrasted between the shallow and deep depths in each of the heated and control bays. In the heated bay (Figure 4), the archaea family BA1 (Bathyarchaeia) that has diverse carbon metabolisms including C1 metabolism (Vanwonterghem et al. 2016) had a significantly increased relative abundance from 8 to 24 cm (*p* < 0.05) as compared to the 0–1 cm surface sediment. In addition, Methanomethylophilaceae, as a methanogenic archaeal family (Borrel et al. 2023), had significantly decreased abundance from 8 to 24 cm compared to the sediment surface (*p* < 0.05). Furthermore, the archaea family EX4484‐6 (Thermoplasmatota) had a clear decreasing abundance from surface to deeper sediment between 5 and 24 cm (all depth ranges *p* < 0.05). In contrast, the family HEL‐GB‐A (Helarchaeales) had significantly increased abundance from 8 to 24 cm as compared to the 0–1 cm sediment. The family JACRNV01 (Thermoplasmatota, SG8‐5 (Yin et al. 2022)) also had significantly increased abundance from 8 to 17 cm. For the bacteria community, the 34–128 family (Atribacterota) significantly increased in abundance compared to the sediment surface as compared to 8–24 cm depths (all depth ranges *p* < 0.05). In addition, the cyanobacteria families Coleofasciculaceae and Cyanobiaceae had significantly decreased abundance (*p* < 0.05 at 15–17 cm and 22–24 cm) as compared to the sediment surface. Finally, the two Desulfbacteria families had different patterns, with the Desulfocapsaceae showing significantly lower abundance at 22–24 cm depth compared to the sediment surface while the UBA11574 family has significantly increased abundance at 8–10 cm compared to the sediment surface (all *p* < 0.05).

In the control bay (Figure 4), the community differences between the surface and deep microbial families were generally less pronounced. For the archaea, the BA1 family only showed a significantly increased relative abundance at 22–24 cm depth as compared to the 0–1 cm layer (*p* < 0.05). In addition, the Methanomethylophilaceae was significantly decreased at 22–24 cm depth as compared to the sediment surface (*p* < 0.05). Furthermore, the Nitrosopumilaceae archaea family was significantly decreased from 15 to 24 cm as compared to the sediment surface (*p* < 0.05). For the bacteria communities, the 34–128 family was significantly increased at 15–17 and 22–24 cm as compared to the sediment surface (both *p* < 0.05). In addition, two families JAAYZQ01 and UBA4823 from class Anaerolineae were also both significantly increased at 15–17 and 22–24 cm depths (all *p* < 0.05). In contrast, the Desulfocapsaceae family had significantly lower abundance at 15–17 and 22–24 cm depths compared to the sediment surface (both *p* < 0.05).

### 3.4 Differential Abundance of Predicted Gene Functions

**FIGURE 5:**
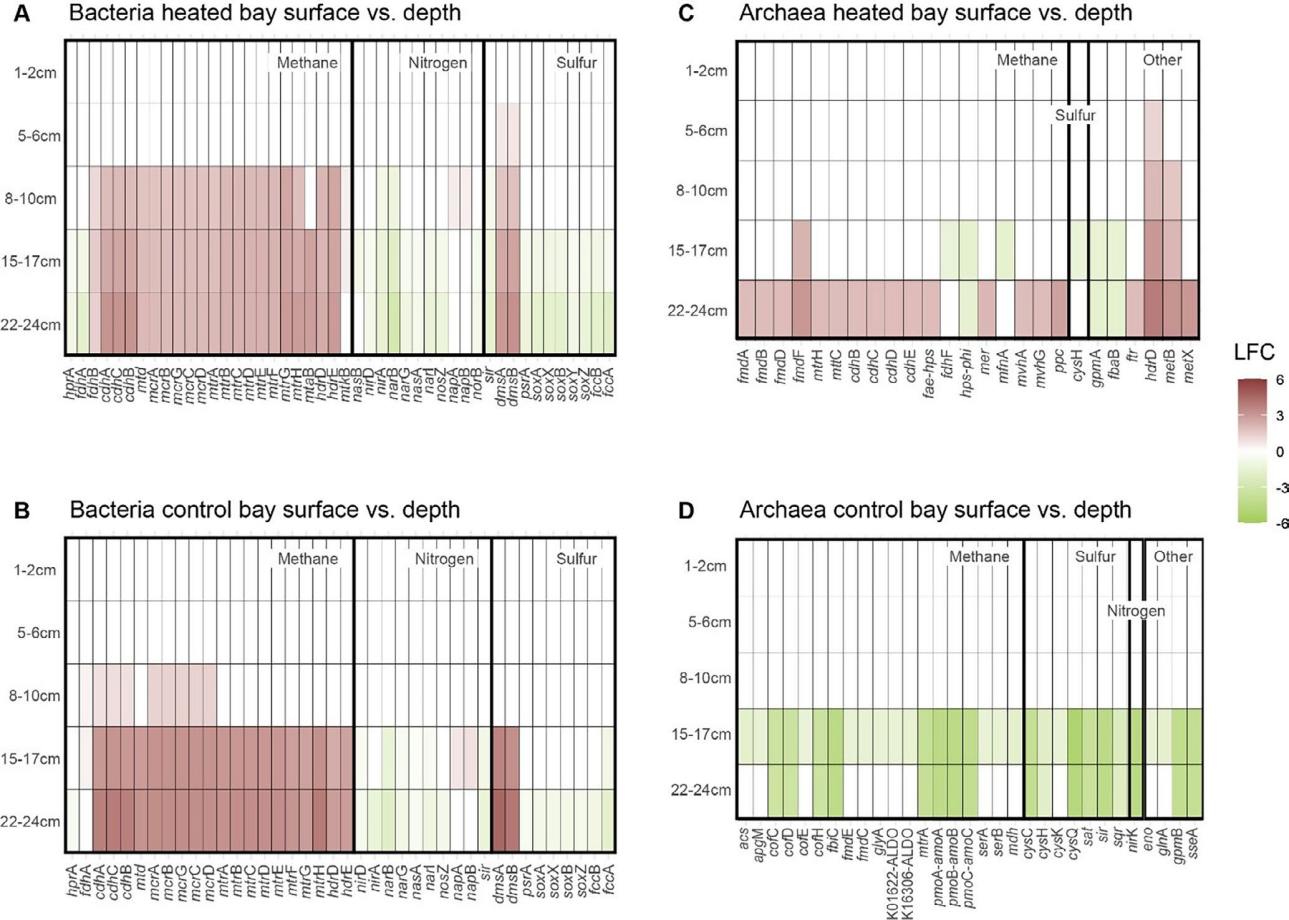
Heated and control bay combined total 16S rRNA gene based PiCRUSt2 analysis. Shown are the significant differential abundant predicted genes within bacteria (A + B) and Archaea (C + D). The surface layers (0–1 cm) in each bay were compared with the deeper layers. Genes of interest (energy metabolism) were filtered, and the log fold change (LFC) is shown with red colours showing higher relative abundances in the deeper sediment layers compared to the surface layers, while the green LFC show lower relative abundance in the deeper layers compared to the surface.

Genes predicted by PICRUSt2 to be involved in nitrogen, sulfur, and methane cycling were selected for analysis with results of genes showing statistically significant differences as compared to the 0–1 cm surface layer (Figure 5). In general, for bacteria and archaea in both bays, few statistically significant differences were observed for the selected gene categories between the baseline 0–1 cm and the sediment slices taken at 1–2 and 5–6 cm depths.

Statistically significant differences were observed for the heated bay 8–10 cm depth zone with increased log fold change (LFC) of genes related to methanogenesis (e.g., *mcrABCD* LFC 1.73 (8–10 cm) to 2.03 (22–24 cm) and *mtrABCDEFGH* LFC 1.68 (8–10 cm) to 2.95 (22–24 cm)) in the deeper layer compared to the surface layer (Figure 5) and also increased LFC of the dimethyl sulphide genes of the sulfur cycle. These predicted gene copy numbers were also significantly higher in the deeper layers of the heated bays, while most nitrogen cycling and oxidative sulfur cycling genes such as SOX coding genes (e.g., *soxA* LFC −0.92 to −1.71 15–24 cm and *soxB* −0.97 to −1.78 15–24 cm) had significantly lower abundance in the deeper layers, compared to the surface layers (0–1 cm). A similar pattern could be observed within the control bay starting at deeper sediment layers from 15 up to 24 cm, showing overall similar predicted gene abundances but starting to decrease at different depths in the two bays.

A different observation was made for the predicted archaea genes (Figure 5). Overall, significant higher relative abundances of genes related to methane cycling were found in the heated bay in the 22–24 cm lowest depth (LFC 1.93–3.05) compared to the surface. While on the other hand, predicted archaeal genes related to for example, methane and sulfur cycling were significantly lower within 15–17 cm with, e.g., methane (LFC −1.44 to −4.40) and 22–24 cm (e.g., methane LFC −3.42 to −4.30) depths compared to the surface layers (0–1 cm) in the control bay.

### 3.5 Geochemical Parameters Relationship to 16S rRNA Gene Based Microbial Community

Average (*n* = 4 ± SD for all) temperature, pH, and salinity of the sampled sites per bay were for the control bay 4.9°C ± 0.4°C, pH 8.48 ± 0.07, and 10.89 ± 0.65 mS/cm (milli Siemens/cm, salinity), respectively. The averages for the heated bay were 7.3°C ± 1°C, pH 8.32 ± 0.25 and 11.64 ± 0.03 mS/cm (File S1). There was a significant difference between the bottom water temperature of each bay (ANOVA, *F*_1,6_ = 21.45, *p* = 0.003). Furthermore, sulfate, phosphate, nitrate, ammonium, organic matter, and dissolved inorganic carbon were measured within porewaters of the sediment depth gradient (File S6). Sulfate showed no significant difference between bays overall but had significant differences between bays along the sediment depth gradient (ANOVA, depth and bay interaction, *F*_5,29.1_ = 4.69, *p* = 0.002). Another variable showing significant differences between bays was dissolved inorganic carbon (ANOVA, *F*_5,5.82_ = 14.32, *p* = 0.009). No other geochemical parameters showed significant differences between bays or along the depth gradient (File S6).

## 4 Discussion

Increased temperature, such as due to climate change, results in higher microbial metabolic rates that in turn, leads to elevated cell replication rates (Clarke and Fraser 2004). Despite this, the qPCR‐based analysis did not show increased cell numbers in relation to higher temperatures in the heated bay in comparison to the control bay. On the contrary, despite having over 50 years to adapt to the increased temperatures, prolonged warming showed fewer microbial cell numbers in the heated bay compared to the control bay. RNA transcript‐based activities have previously demonstrated increased energy production along with increased RNA transcript counts for e.g., chaperones observed in the heated bay (Seidel, Ketzer, et al. 2022). Therefore, the decreased heated bay cell numbers may be related to a portion of the available energy being diverted to combating stress, as opposed to being used for replication. This was further supported in a mesocosm experiment modelling climate change related ocean acidification that showed decreased heterotrophic production and bacterial cell numbers (Bunse et al. 2016) and a decrease in cell numbers coupled to an increased temperature due to stress and nutrient availability in soil and freshwater lake sediment (Haglund et al. 2003; Williams 2007). In addition, microbial abundances also generally decrease with sediment depth due to decreased availability of electron donors and less efficient energy conservation coupled with anaerobic respiration (Kallmeyer et al. 2012). Therefore, the more rapid decrease in cell numbers in the heated bay that may also be coupled with anaerobic reactions occurring closer to the heated bay sediment surface, i.e., the shallowing of geochemical zones (Seidel, Ketzer, et al. 2022; Seidel, Broman, et al. 2023). In summary, higher temperatures increase metabolic rates in microorganisms but this did not translate into an increase in heated bay bacterial cell numbers. This suggested that despite the elevated metabolic rates associated with warming, climate change may impact microbial cell numbers, and the resultant biochemical processes they perform, with potential cascading effects on the entire ecosystems food web.

The PMA‐related investigation of live and dead cells showed no statistical difference between the PMA(+) and PMA(−) samples in either of the two bays. Although this might be a valid result, the high degree of variation, particularly in the control bay PMA(−) samples, may have masked any differences. The lack of difference in the total number of live cells may be due to the fact that important contributors to bacterial mortality—such as grazers and viruses—do not play a significant role in sediments, resulting in a higher proportion of consistently active cells (Haglund et al. 2003). While in this study the deepest sediment samples analysed were collected from depths of less than 0.5 m, virus‐induced bacterial mortality typically increases with sediment depth, with large viral abundances reported up to 1 m below the marine sediment surface (Mei and Danovaro 2004). A further potential reason for the lack of differences between the PMA(+) and PMA(−) samples could have been the above‐mentioned higher metabolic rate due to temperature increases and the use of dead cell material (also termed ‘necromass’) for energy production (Hirakata et al. 2023) in the heated bay. Microbial communities in the deep marine subsurface are a selection of rare (low relative abundance) species present in the sediment surface that are selected during burial (Starnawski et al. 2017) in a process that starts with a shift in a marine sediment community below the depth of bioturbation at approximately a depth of 7 cm from the surface (Petro et al. 2019). The use of necromass is an important energy source in anoxic groundwaters, with a similar lack of difference between live and dead cells as observed in this study (Lopez‐Fernandez et al. 2018) and despite the differences in systems, a similar efficient use of necromass in the Baltic Sea sediment may have occurred. In addition, the PMA treatment protocol for untreated marine sediment samples containing sediment particles may require specific adjustments, such as increasing the PMA concentration and incubation time (Nocker et al. 2007). The method used in this study was based upon the use of PMA in Baltic Sea sediments sampled from a different location (Broman, Sachpazidou, et al. 2017). In the earlier study, the PMA based data were supported by RNA transcripts showing the dominant populations in the PMA(+) samples were indeed active. Furthermore, although PMA treatment is applicable to both Gram‐positive and Gram‐negative bacteria (Nocker et al. 2006), its efficiency can vary due to differences in cell wall structure. In this study, with the exception of the Ilumatobacteraceae that may have a different efficiency, the bacterial communities were dominated by Gram‐negative taxa suggesting that the susceptibility to PMA was likely consistent across the Gram negative dominant taxa.

Both bacterial and archaeal 16S rRNA gene amplicon results agreed with previous findings in the two bays showing modification of the microbial community with depth (Seidel, Sachpazidou, et al. 2023). In general, differences in taxa between sediment layers were greater with warmer temperatures, especially within deeper layers. This was particularly true for some methanogens and sulfate reducers showing significant abundance changes closer to the sediment surface within long‐term warmer coastal waters. For example, the hydrogenotrophic methanogen BA1 (Maus et al. 2016) was increased at 1–2 cm below sediment surface in the heated bay as compared to a sediment depth of 15 cm in the control bay. These data supported the SMTZ shifting closer towards the sediment surface during the overall shallowing of the geochemical layers (Seidel, Ketzer, et al. 2022). This shallowing of the SMTZ closer to the sediment surface in coastal regions increases the chance of methane escaping through diffusion and entering the water column (Egger et al. 2018). This is supported by long‐term warming experiments (average of 4°C) in freshwater ponds showing increased methane emissions (Zhu et al. 2020), a shift to increased methanogens being favoured by warmer conditions (Blake et al. 2020), and increased relative abundances of sulphate‐reducing bacteria closer to the sediment–water interface due to prolonged warming (Sawicka and Brüchert 2017; Seidel, Ketzer, et al. 2022; Seidel, Sachpazidou, et al. 2023). The syntrophic relationship of sulfate‐reducing bacteria and archaea could potentially be linked to higher sulfate fluxes (Hinrichs and Boetius 2003) and microbial mediated sulfate reduction is closely linked to temperature. Overall, the shallower 16S rRNA gene‐based identification of methanogens and sulfate reducers in the heated bay confirmed a shift of the SMTZ closer to the sediment–water interface with an increased risk of methane release to the benthic water (Seidel, Ketzer, et al. 2022).

Combining the 16S rRNA gene‐based taxonomy with quantitative cell numbers showed no clear pattern in the heated bay coastal sediments. However, higher abundances of the archaea EX4484‐6 (Thermoplasmatota) were observed in the heated bay surface sediments that only occurred in higher abundances at deeper depths of the control bay. The Thermoplasmatota are methanogenic archaea (Garcia et al. 2000) that utilise detrital organic matter within sediments (Zheng et al. 2022). This further supports the shallowing of geochemical layers, higher microbial activity in the upper sediment layers, and a shift of microbial communities with prolonged warming. In contrast to the lack of clear patterns in the heated bay, a clear dominance of Coleofasciculaceae (bacteria) and TCS64 (archaea) was observed in the ambient control bay. The high abundance of the cyanobacteria supports previous results (Seidel, Ketzer, et al. 2022) with the Coleofasciculaceae family being mostly found in terrestrial and intertidal zones potentially from freshwater input (Fernandes et al. 2021). The most abundant archaea within the control bay sediment were the uncultured TCS64 *Can*. Bathyarchaeota with increasing abundances with sediment depth, supporting previous reports that it is one of the dominant groups found in marine sediment communities (Kubo et al. 2012). One reason for its higher abundance in especially the surface sediment can be linked to the methane metabolism (Lloyd 2015) and the shift of the SMTZ as those archaea are typically found in higher abundances along surface depths (Webster et al. 2011). Finally, the PICRUSt2 based predictions of the potential functions and energy consumption sources once again supported the shift in microbial community seen in the 16S rRNA gene amplicon data. One important difference was the predicted electron acceptors significantly changing from nitrogen to sulphur and finally methane at a shallower depth in the heated bay sediment compared to control bay sediment. All these data support previous studies showing increased RNA gene transcript‐based expression for sulfur and methane energy cycling closer to the sediment surface as a result of prolonged and increased warming (Perner et al. 2022; Seidel, Ketzer, et al. 2022).

## Author Contributions

**Laura Seidel:** designed the study, collected plus analysed the data and wrote – original draft. **Songjun Li:** collected plus analysed the data and wrote – original draft. **Shahinez Hanna‐Elias:** collected plus analysed the data and wrote – review and editing. **Iryna Rula:** collected plus analysed the data and wrote – review and editing. **Louise Ahlberg:** analysed the data and wrote – review and editing. **Anders Forsman:** wrote – review and editing. **Samuel Hylander:** wrote – review and editing. **Marcelo Ketzer:** wrote – review and editing. **Mark Dopson:** designed the study and wrote – original draft.

## Funding

This work was supported by Svenska Forskningsrådet Formas, FR‐2022‐01016, FR‐2020/01338.

Vetenskapsrådet, 2020‐03519.

## Conflicts of Interest

The authors declare no conflicts of interest.

## Supporting information


**File S1:** Details of sampling sites, environmental variables, 16S rRNA gene sequencing plus count data, and qPCR analyses.
**File S2:** 16S rRNA gene rarefaction (A, B), Shannon's H index analyses (C, D) and statistics table.
**File S3:** Statistics of 16S rRNA gene sequencing data between the PCR amplifications in the presence and absence of propidium monoazide.
**File S4:** Differential abundance analyses on the 16S rRNA gene amplicon data at the different sediment depths. Found on: https://github.com/laseab/PMA_depth_bays.
**File S5:** Raw output from the PiCRUST2 16S rRNA gene metabolic predictions. Found on: https://github.com/laseab/PMA_depth_bays.
**File S6:** Selected environmental variables along the depth profile within the heated and control bays.

**Data S1:** emi70256‐sup‐0002‐DataS1.xlsx.

## Data Availability

The raw sequencing data is available online at European nucleotide archive (https://www.ebi.ac.uk/ena) under the project accession number: PRJEB85159 and sample accession numbers from ERS23014956 to ERS23015143. The R script detailing the analysis can be downloaded at https://github.com/laseab/PMA_depth_bays.
