# Supplementary material for: Warming Causes a Decline in Baltic Sea Coastal Sediment Microbial Abundance
Source: Environ Microbiol. 2026 Feb 19;28(2):e70256. doi: 10.1111/1462-2920.70256 (PMC12920023; doi:10.1111/1462-2920.70256)
Supplement: Supplementary file 1 — File S1: Details of sampling sites, environmental variables, 16S rRNA gene sequencing plus count data, and qPCR analyses. File S2: 16S rRNA gene rarefaction (A, B), Shannon's H index analyses (C, D) and statistics table. File S3: Statistics of 16S rRNA gene sequencing data between the PCR amplifications in the presence and absence of propidium monoazide. File S4: Differential abundance analyses on the 16S rRNA gene amplicon data at the different sediment depths. Found on: https://github.com/laseab/PMA_depth_bays. File S5: Raw output from the PiCRUST2 16S rRNA gene metabolic predictions. Found on: https://github.com/laseab/PMA_depth_bays. File S6: Selected environmental variables along the depth profile within the heated and control bays. [file EMI-28-e70256-s002.docx]

**Climate-Induced Warming Triggers Sharp Decline in Microbial Abundance in
Baltic Sea Coastal Sediments**

Laura Seidel, Songjun Li, Shahinez Hanna-Elias, Iryna Rula, Louise Ahlberg,
 Anders Forsman, Samuel Hylande^1^, Marcelo Ketzer, Mark Dopson

**Supplementary File S1.** Sampling sites, environmental variables, 16S rRNA gene sequencing plus count data, and qPCR analyses.

The data can be found in the separate Excel File.

**Supplementary File S2.** 16S rRNA gene rarefaction (A, B), Shannon’s H index analyses (C, D) and statistics table. Shannon´s H alpha diversity of control and heated bay with and without PMA in bacteria (C) and archaea (D); green without PMA and red with PMA. Statistical analysis on alpha diversity data – mixed linear model using lme() function in R with depth, bay, and PMA as fixed variables with interaction and site as random variable is shown in the table below.


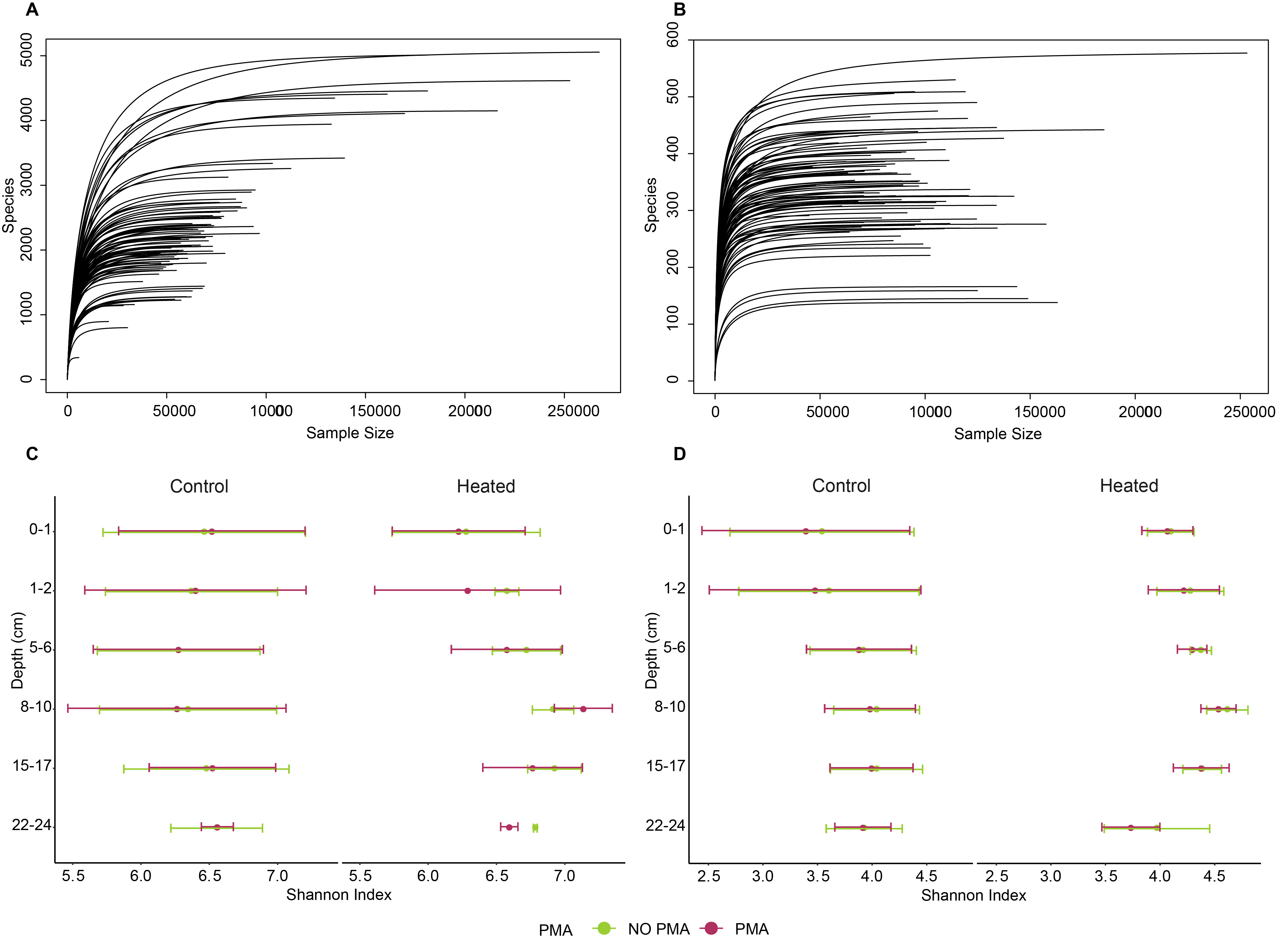


| **Organism** | **Variable** | **numDF** | **denDF** | **F-value** | **p-value** |
| --- | --- | --- | --- | --- | --- |
| Archaea | depth (num) | 1 | 80 | 4.42 | 0.03 |
|  | bay | 1 | 6 | 6.43 | 0.04 |
|  | PMA | 1 | 80 | 0.75 | 0.39 |
|  | depth*bay | 1 | 80 | 4.84 | 0.03 |
|  | Depth*PMA | 1 | 80 | 0.004 | 0.94 |
|  | Bay*PMA | 1 | 80 | 0.0006 | 0.98 |
|  | depth*bay*PMA | 1 | 80 | 0.21 | 0.64 |
| Bacteria | depth(num) | 1 | 80 | 11.08 | 0.0013 |
|  | bay | 1 | 6 | 0.62 | 0.46 |
|  | PMA | 1 | 80 | 0.46 | 0.50 |
|  | Bay*depth | 1 | 80 | 5.911 | 0.01 |
|  | Depth*PMA | 1 | 80 | 0.0002 | 0.98 |
|  | Bay*PMA | 1 | 80 | 0.68 | 0.41 |
|  | depth*bay*PMA | 1 | 80 | 0.03 | 0.85 |

**Supplementary File S3.** Statistics of 16S rRNA gene data between the PCR amplifications in the presence and absence of propidium monoazide.

| **method** | **Variable** | **n** | **statistic** | **df** | ***p*** | **Effect size** |
| --- | --- | --- | --- | --- | --- | --- |
| friedman | bacteria | 47 | 0.19 | 1 | 0.66 | 0.004 |
| friedman | archaea | 47 | 1.04 | 1 | 0.30 | 0.022 |

**Supplementary File S4.** Differential abundance analyses on the 16S rRNA gene data at the different sediment depths.

Found on: <https://github.com/laseab/PMA_depth_bays>

**Supplementary File S5.** PiCRUST2 raw output data for the 16S rRNA gene ASV predictions.

Found on: <https://github.com/laseab/PMA_depth_bays>

**Supplementary File S6.** Depth profiles of selected environmental variables within the heated and control bays.

**
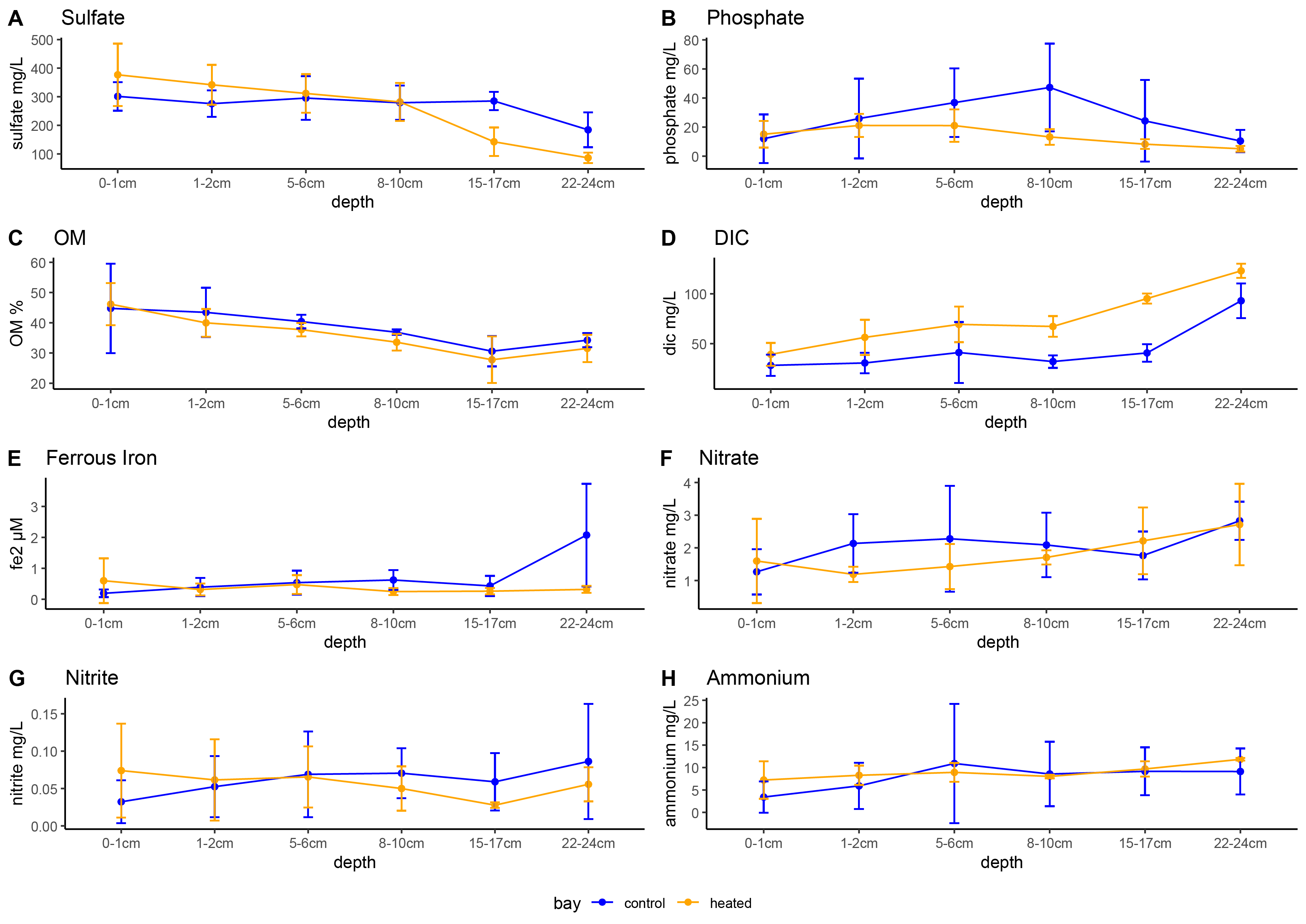
**
